# Surgical outcome and prognostic factors in epilepsy patients with MR-negative focal cortical dysplasia

**DOI:** 10.1371/journal.pone.0249929

**Published:** 2021-04-14

**Authors:** Min Jae Seong, Su Jung Choi, Eun Yeon Joo, Young-Min Shon, Dae-Won Seo, Seung Bong Hong, Seung Chyul Hong

**Affiliations:** 1 Department of Neurology, Myongji Hospital, Hanyang University College of Medicine, Goyang, Korea; 2 Department of Clinical Nursing Science, Graduate School of Clinical Nursing Science, Sungkyunkwan University, Seoul, Korea; 3 Department of Neurology, Neuroscience Center, Samsung Medical Center, Sungkyunkwan University School of Medicine, Seoul, Republic of Korea; 4 Department of Neurology, Samsung Medical Center, Sungkyunkwan University School of Medicine, Seoul, Republic of Korea; 5 Department of Neurosurgery, Samsung Medical Center, Sungkyunkwan University School of Medicine, Seoul, Republic of Korea; Cook Children\’s Health Care System: Cook Children’s Medical Center, UNITED STATES

## Abstract

**Objective:**

Focal cortical dysplasia (FCD) represents a heterogeneous group of disorders of the cortical formation and is one of the most common causes of epilepsy. Magnetic resonance imaging (MRI) is the modality of choice for detecting structural lesions, and the surgical prognosis in patients with MR lesions is favorable. However, the surgical prognosis of patients with MR-negative FCD is unknown. We aimed to evaluate the long-term surgical outcomes and prognostic factors in MR-negative FCD patients through comprehensive presurgical data.

**Methods:**

We retrospectively reviewed data from 719 drug-resistant epilepsy patients who underwent resective surgery and selected cases in which surgical specimens were pathologically confirmed as FCD Type I or II. If the epileptogenic focus and surgical specimens were obtained from brain areas with a normal MRI appearance, they were classified as MR-negative FCD. Surgical outcomes were evaluated at 2 and 5 years, and clinical, neurophysiological, and neuroimaging data of MR-negative FCD were compared to those of MR-positive FCD.

**Results:**

Finally, 47 MR-negative and 34 MR-positive FCD patients were enrolled in the study. The seizure-free rate after surgery (Engel classification I) at postoperative 2 year was 59.5% and 64.7% in the MR-negative and positive FCD groups, respectively (p = 0.81). This rate decreased to 57.5% and 44.4% in the MR-negative and positive FCD groups (p = 0.43) at postoperative 5 years. MR-negative FCD showed a higher proportion of FCD type I (87.2% vs. 50.0%, p = 0.001) than MR-positive FCD. Unilobar cerebral perfusion distribution (odds ratio, OR 5.41) and concordance of interictal epileptiform discharges (OR 5.10) were significantly associated with good surgical outcomes in MR-negative FCD.

**Conclusion:**

In this study, MR-negative and positive FCD patients had a comparable surgical prognosis, suggesting that comprehensive presurgical evaluations, including multimodal neuroimaging studies, are crucial for obtaining excellent surgical outcomes even in epilepsy patients with MR-negative FCD.

## Introduction

Approximately one-third of patients considering surgical treatment for drug-resistant epilepsy show a normal magnetic resonance imaging (MRI) appearance, that is called MR-negative epilepsy. The presence of lesions on MRI is an essential factor in predicting the surgical results [1–4]. A meta-analysis revealed that the seizure-free rate was 2.5 times higher in MR-positive epilepsy than MR-negative epilepsy [4]. As a result, physicians are reluctant to decide on conducting an epilepsy surgery even in intractable epilepsy patients with normal MRI.

Focal cortical dysplasia (FCD) is the most common etiology of drug-resistant MR-negative epilepsy. FCD is a subgroup of cortical malformations characterized by an aberrant cortical organization resulting from abnormal neuronal migration and differentiation [5]. It includes pathologic features such as neuronal heterotopias, dyslamination, and a combination of bizarre pyramidal neurons of the cerebral cortex and white matter and the presence of balloon cells [6]. FCD accounts for 20–25% of focal epilepsy [7,8], and 25–40% of childhood drug-resistant epilepsy cases are FCD [9].

Several studies involving FCD-proven surgery cases showed that young age at surgery, shorter duration of epilepsy, unilobar localization of the epileptic focus, extended resection, and lesion localization in the temporal lobe were good prognostic factors in adults [10–12]. Poor prognostic factors include incomplete resection, secondarily generalized tonic-clonic seizures (GTCS), later epilepsy onset, multilobar extension, longer epilepsy duration, the need for intracranial EEG recordings and FCD Type I [3,12–14]. A study of pediatric patients reported MRI lesions and FCD Type II as good prognostic factors [1].

Multimodal neuroimaging techniques play a role in the delineation of the epileptic foci in the presurgical evaluation. The co-registration of MRI with positron emission tomography using 2-deoxy-2-[fluorine-18] fluoro-D-glucose (FDG-PET) improve the diagnostic accuracy and surgical prognosis in MR-negative FCD patients [15]. Voxel-based morphometric MRI post-processing techniques or cortical feature analysis, and machine learning were adopted to detect subtle epileptogenic structural lesions in MR-negative FCD patients [16,17].

Nevertheless, there are scarce data on the surgical prognosis and favorable or unfavorable prognostic factors in epilepsy patients with MR-negative FCD. We hypothesized that the surgical outcome of epilepsy patients with MR-negative FCD would not be worse than that of MR-positive FCD patients if comprehensive presurgical evaluations were performed to determine the epileptic foci and delineate the resection margin even in MR-negative FCD patients. To test this hypothesis, we assessed the short- and long-term surgical outcomes and evaluated prognostic factors by comparing clinical information, electrophysiological findings as well as structural and functional neuroimaging studies between MR-negative and MR positive FCD patients.

## Methods

### Ethical publication statement

All patients provided a written informed consent for participation in the study. Written informed consent was obtained from the next of kin, caretakers, or guardians on behalf of the minors/children participants involved in this study. The study was approved by the Institutional Review Board of the Samsung Medical Center. We confirm that we have read the Journal’s position on issues pertaining to ethical publication and affirm that this report is consistent with those guidelines

#### 1. Patients

We reviewed a database of 719 patients with drug-resistant epilepsy who underwent resective epilepsy surgery at a university-affiliated hospital between January 2001 and May 2018. We selected patients whose surgical specimens were shown to have FCD Type I or II by histopathological examination. Patients with hippocampal sclerosis, tumor, vascular anomaly, and FCD type III were excluded from the study.

A total of 81 patients with histologically confirmed FCD Type I or II were selected out of 719 cases. The patients were divided into two groups: (non-lesional) MR-negative FCD (n = 47) and (lesional) MR-positive FCD (n = 34).

#### 2. Presurgical comprehensive evaluation

It consisted of a thorough neurological examination, ictal and interictal EEG monitoring, and brain MRI during the first admission period. Ictal and interictal single-photon emission computed tomography (SPECT) was performed to lateralize or localize the epileptic foci [18–20]. Each patient underwent FDG-PET, a neuropsychological test, and, if needed, the Wada test during the second admission [21]. All data were reviewed and discussed in an epilepsy management conference at which the surgical strategy was discussed.

#### 2.1 Analysis of clinical seizures during scalp video EEG monitoring

We carefully reviewed each patient’s seizures. The presence of an aura was determined by the patient’s memory or the patient pressing a button before seizures.

**Scalp video EEG monitoring (sEEG)** A 10/10 system was used for scalp electrodes. AEDs were usually reduced or stopped to facilitate the recording of seizures.

**Interictal EEG classification** Interictal epileptiform discharges (IEDs) were counted and analyzed the over entire recording period and classified into four types: 1) regional (single lobe or contiguous region), and non-regional (multilobar, hemispheric, generalized), 2) concordant and discordant. IEDs were defined as regional when there was a 75% or more preponderance in one lobe or contiguous regions and as non-regional when present in ≥ two non-contiguous regions with less than 75% preponderance in any single lobe. Discordant was defined as the discrepancy between the IED distribution and resection area.

**Ictal EEG classification during scalp EEG recording** Habitual seizures were recorded at least three times for ictal EEG analysis. Regional was diagnosed when the location of the scalp ictal EEG onset (sEEG onset) was confined to one lobe or contiguous regions or when the amplitude ratio of one lobe or contiguous regions versus the other lobes was greater than 2:1 in bipolar montages and greater than 2:1 for the two sides in the referential montages. Non-regional was diagnosed when the sEEG onset initiated from ≥ 2 non-contiguous lobes over both hemispheres independently or synchronously. Bilateral onset (nonlateralized) was defined as a simultaneous ictal onset pattern in both hemispheres. Discordant was diagnosed when there was a discrepancy between the scalp ictal EEG onset and the resection area. Ictal EEG onset patterns were classified in detail as follows: 1) rhythmic activity, 2) paroxysmal fast, 3) suppression, and 4) repetitive epileptiform activity.

### Intracranial EEG (iEEG) monitoring

All patients involved in this study underwent iEEG monitoring using a combination of grids/strips with or without depth electrodes. Anatomical targeting of electrodes was established in each patient according to available non-invasive information and hypotheses regarding the localization of the epileptogenic zones. The ictal onset zone (IOZ) was identified as any paroxysmal, sustained ictal EEG pattern during the iEEG monitoring that was distinct from background activity and accompanied by clinical seizures [22].

#### 2.2 Neuroimaging studies

*Brain MRI*. MRI was performed using a GE Signa 1.5-Tesla scanner (GE Medical System, Inc., Milwaukee, WI, USA) or a 3.0-Tesla scanner (Philips, Best, The Netherlands). MRI was performed with the epilepsy protocol, including spoiled gradient echo; T1-weighted coronal, axial, and sagittal planes; T2-oblique coronal and axial plane imaging; and fluid-attenuated inversion recovery. An experienced neuroradiologists and physicians determined which MRI had gyration anomalies, focal thickening of the cortex, blurring of the gray-/white-matter junction, and abnormal cortical and subcortical signal intensity consistent with a diagnosis of FCD [8]. MRI diagnosis was confirmed after discussing the patients’ management conference in which epileptologists and neuroradiologists participated.

*FDG-PET studies*. FDG-PET was performed in the interictal period during which there were no seizures for more than 24 h to confirm the hypometabolism region. The details have been described in previous studies [21,23].

*Interictal and ictal SPECT studies*. Brain SPECT was performed after 30–60 min by injecting 25 mCi 99mTc-ethyl cysteinate dimer and using a 3-headed Triad XLT system (Trionix Research Laboratory, Inc., Twinsburg, OH). Interictal and ictal SPECT as subtraction Ictal SPECT Co-registered to MRI (SISCOM) were performed. The radiotracer injection time in the SISCOM was checked. A detailed analysis has been described in previous literature [19].

#### 3. Surgery and outcome

The surgery was classified into temporal, frontal, parietal, occipital, and multilobar regions according to location. Complete resection occurred when resection margins included the IOZ with or without frequent interictal spikes in adjacent brain regions and early ictal propagation on iEEG monitoring. After surgical resection, electrocorticography (ECoG) was recorded around the resection margin to confirm that any spike disappeared completely. When active spikes were observed around the resection margin in the recorded ECoG after resection, the active region was additionally resected [24,25]. The resected specimens were pathologically classified into FCD Type I or II based on Blumcke’s classification [26].

All patients had a minimum follow-up period of two years and a maximum of 18 years. When the IOZ was diffuse (non-localized) or included eloquent areas within the resection margins, incomplete resection was performed. Patients were classified as seizure-free if they achieved Engel classification I by the last year of follow-up. The patients were instructed to visit the clinic 1 month after surgery and then every 3 months. If patients became seizure-free, hospital visits were scheduled every 6 months. Postoperative seizure frequency and possible provocative factors were documented. Surgical outcomes were evaluated at 2 and 5 years after surgery. Postoperative seizure outcomes were determined by outpatient clinic or telephone interviews using Engel’s classification. The seizure-free rate was assessed within a five-year follow-up period.

#### 4. Statistical analysis

Continuous variables were expressed as mean ± standard deviation (SD), and categorical data were expressed as percentages and numbers. All continuous variables were analyzed using independent t-test or Mann-Whitney U-test, and categorical variables were analyzed using the Fisher’s exact test or chi-square test. Univariate and multivariate regression analyses were performed to evaluate the effect of the variables on postoperative surgical outcome. Multivariate Cox proportional hazards regression analysis was assessed as a variable with a p-value < 0.2, in the univariate analysis. The seizure-free rate was compared by Kaplan-Meier survival analysis, and prognostic factors were evaluated by log-rank test and Cox proportional hazard regression with variables known to be related to seizure recurrence or, alternatively, any displaying a p-value < 0.2 in univariate analysis. Statistical significance was defined as p < 0.05. Statistical analysis was performed with SPSS software (version 18.0, Chicago, IL, USA).

## Results

### Patients’ characteristics

**Fig 1:**
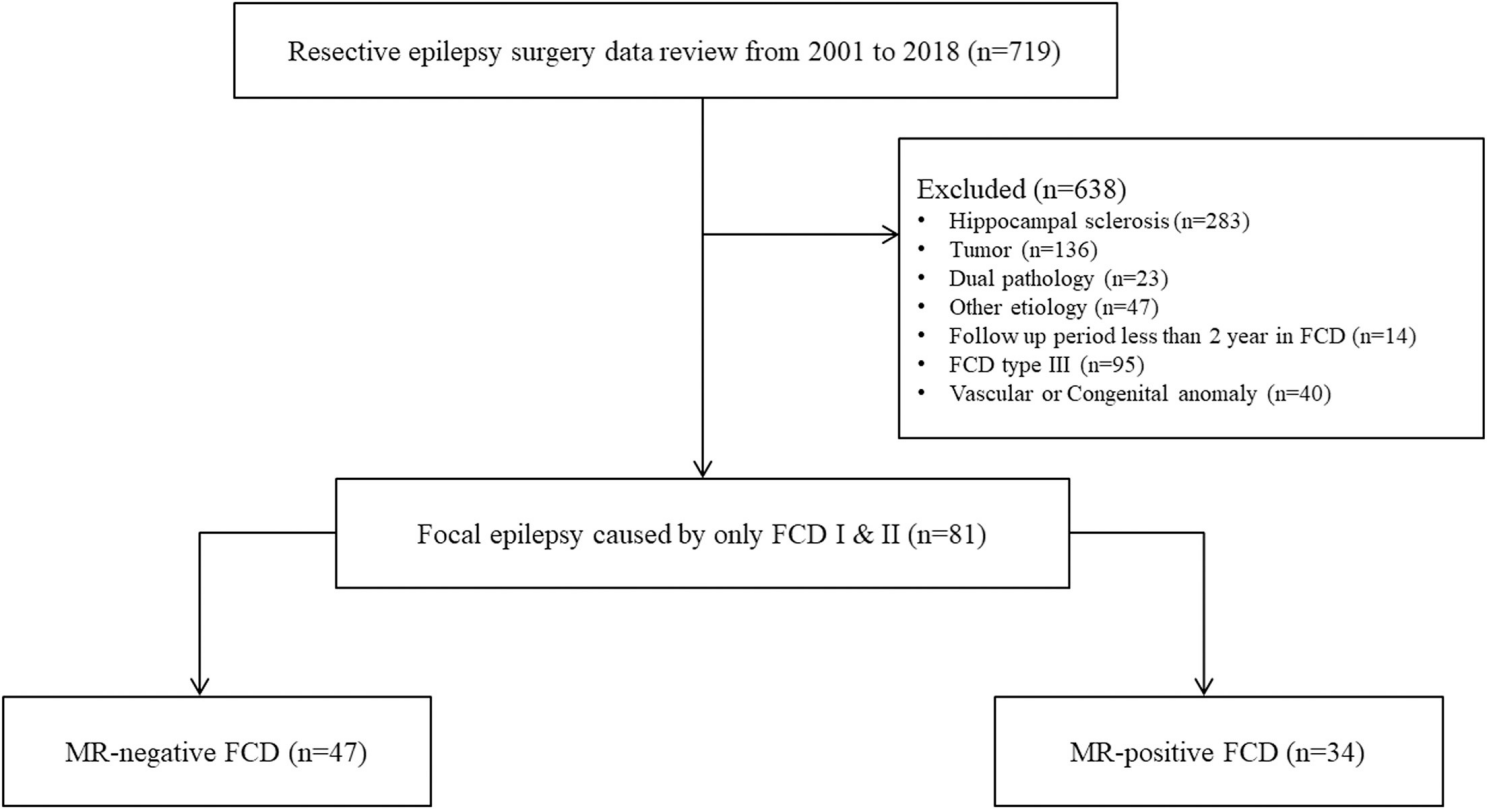
Enrollment log. FCD, focal cortical dysplasia. FCD Type III was defined as FCD Type I adjacent or affecting the same cortical area/lobe to another principal lesion, including hippocampal sclerosis, glial or glioneuronal tumor, vascular malformation, and any other lesion acquired during early life [26].

This study included 81 patients (42 women). Exclusion criteria were patients with hippocampal sclerosis (n = 283), tumor (n = 136), vascular or congenital etiologies (n = 40), dual pathology (n = 23), and others including gliosis or infarction (n = 47) as the etiology of epilepsy. Patients with FCD Type III (n = 95) and those who were followed up for less than 2 years (n = 14) were also excluded (Fig 1).

Mean follow-up period in the present study was 7.7 ± 3.9 years (range 2–18 years) for MR-positive FCD and 6.8 ± 3.1 years (range 2–14 years) for MR-negative FCD patients (p = 0.30, Table 1). The age at seizure onset and surgery was definitely higher, and the proportion of patients with daily seizures was lower in MR-negative FCD than MR-positive FCD patients. GTC history was more frequent in patients with MR-negative FCD (Table 1). The location of the intracranial EEG implantation to identify the epileptogenic zone is as follows: 1) temporo-occipital (TO, n = 9), 2) fronto-temporal (FT, n = 16), 3) temporo-parietal (TP, n = 8), 4) fronto-parietal (FP, n = 7), 5) fronto-temporo-parietal (FTP, n = 9), 6) temporo-parietooccipital (TPO, n = 9), 7) temporal (n = 18), and 8) frontal (n = 5). Electrophysiological data and functional neuroimaging results did not show any significant differences between the MR-negative and MR-positive FCD groups. The most common resected area in MR-negative FCD was the temporal and frontal lobes in MR-positive FCD. The prevalence of FCD Type I was significantly higher than that of FCD Type II in the MR-negative FCD group (89.4% vs. 10.6%), while the proportion of Type I and Type II FCD was the same in MR-positive FCD (50% vs. 50%) (Table 2).

**Table 1:** Demographics.

	MR-negative FCD (n = 47)	MR-positive FCD (n = 34)	*P*
Male (%)	18 (38.3)	21 (61.8)	0.04*
Age at onset, years	16.00 ± 9.69	10.08 ± 9.57	0.008*
Age at surgery, years	31.13 ± 9.87	23.41 ± 13.00	0.003*
Duration of seizure, years	15.09 ± 8.57	13.32 ± 9.63	0.38
Epilepsy duration, >10y (%)	34 (72.3)	18 (52.9)	0.10
Seizure frequency, /m	20.98 ± 52.49	41.39 ± 67.54	0.13
Daily seizure frequency (%)	9 (19.1)	15 (45.4)	0.01*
GTC history (%)	39 (82.9)	17 (50.0)	0.003*
Number of AEDs	3.64 ± 1.29	3.71 ± 1.66	0.83
Mean follow-up period, years	6.8 ± 3.1	7.7 ± 3.9	0.30

Note: Data are presented as mean ± standard deviation, or n (%) values. Chi-square test for categorical variables and student t-test for continuous variables.

Abbreviation: GTC, generalized tonic-clonic seizure; AED, antiepileptic drug.

*p<0.05, independent t-test or Mann-Whitney U-test.

**Table 2:** Results of the presurgical evaluations and surgical outcome.

Variables	Category	MR-negative FCD (n = 47)	MR-positive FCD (n = 34)	*P*
Ictal EEG, scalp	Regional	28 (59.6)	17 (50.0)	0.49
	Lateralized	30 (63.8)	22 (64.7)	0.93
	Concordant	19 (40.5)	17 (50.0)	0.49
Ictal EEG onset classification	Rhythmic activity	33 (70.2)	19 (55.9)	0.27
	Paroxysmal fast	9 (19.1)	9 (26.5)	
	Suppression	0 (0)	2 (5.9)	
	Repetitive epileptiform activity	5 (10.6)	4 (11.8)	
Interictal EEG, scalp	Regional	25 (59.5)	14 (48.3)	0.46
	Concordant	22 (52.3)	20 (68.9)	0.22
PET^†^	Multilobar	22 (46.8)	11 (37.9)	0.48
	Unilobar	25 (53.2)	18 (62.1)	
	Discordant	23 (48.9)	9 (31.0)	0.15
	Concordant	24 (51.1)	20 (69.0)	
SISCOM^††^	Multilobar	19 (47.5)	8 (32.0)	0.30
	Unilobar	21(52.5)	17 (68.0)	
	Discordant	18 (45.0)	10 (40.0)	0.79
	Concordant	22 (55.0)	15 (60.0)	
	Radiotracer injection time (sec)	30.49±24.82	31.21±16.70	0.89
Resective areas based on iEEG implantation	Frontal	7 (14.8)	13 (38.2)	0.03*
	Temporal	23 (48.9)	9 (26.4)	
	Parietal	2 (4.2)	3 (8.8)	
	Occipital	1 (2.1)	3 (8.8)	
	Multilobar	14 (29.7)	6 (17.6)	
Pathology	FCD Type I	42 (89.4)	17 (50.0)	0.001*
	FCD Type II	5 (10.6)	17 (50.0)	
Surgical outcome	At two year	28 (59.5)	22 (64.7)	0.81
(Engel I)	At five year	19 (57.5)	12 (44.4)	0.43

Note: Data are presented as n (%) values. Ictal EEG and interictal EEG, data from scalp video-EEG monitoring.

^†^Number of analyses = MRI negative: MRI positive = 47:29

^††^ Number of analyses = MRI negative: MRI positive = 40:25.

Abbreviations: Discordant, not in agreement with the results from subdural ictal EEG monitoring; Concordant, in agreement with the results from iEEG monitoring; SISCOM, subtraction ictal and interictal SPECT co-registered to MRI; iEEG, intracranial EEG recordings, represent the area where subdural or depth electrodes insertion; FCD, focal cortical dysplasia.

*p < 0.05, Fisher’s exact test or chi-square test.

### Surgical outcome assessment

**Fig 2:**
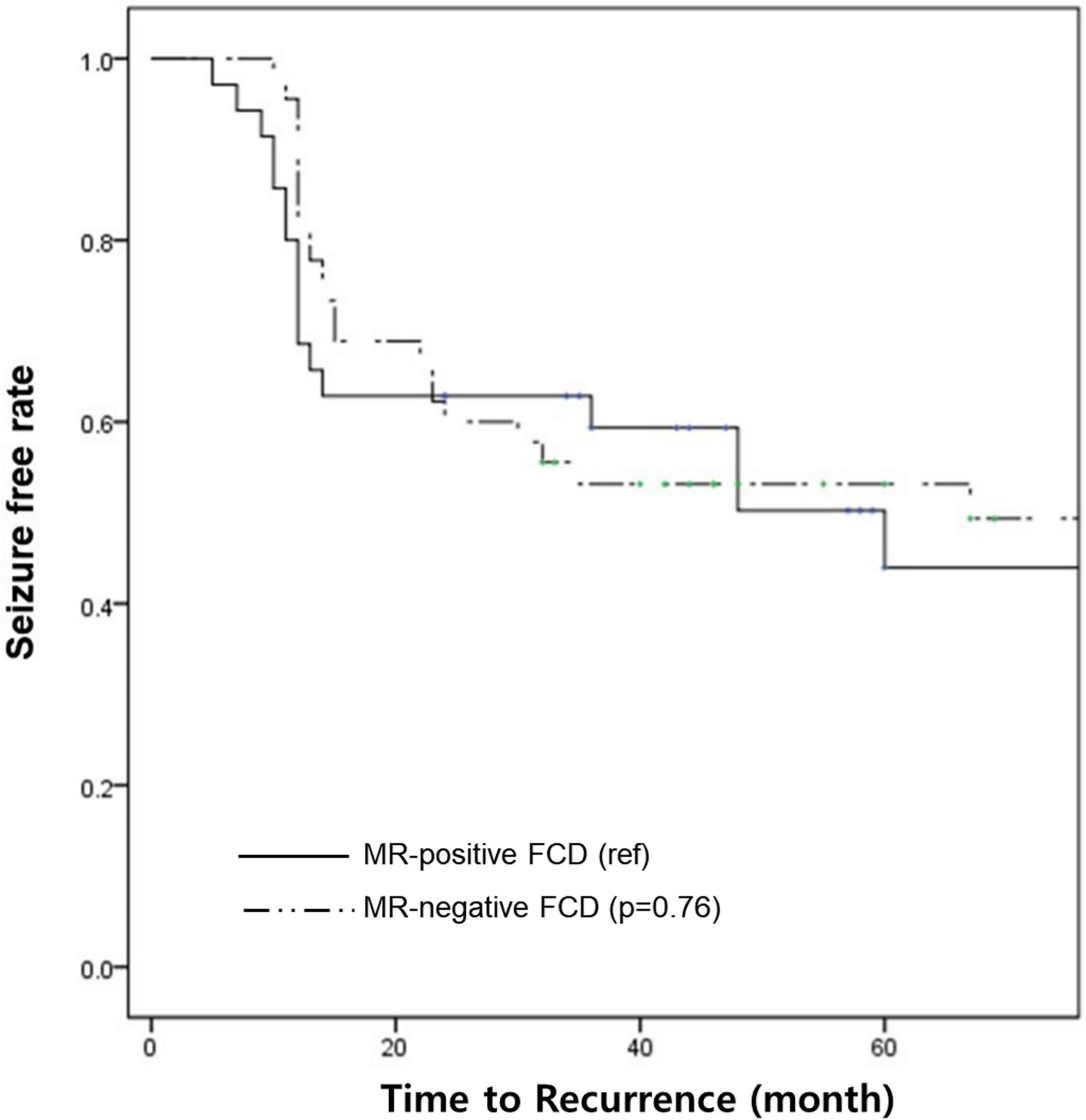
Kaplan-Meier survival curves according to MRI findings. The survival curves showed no statistical significance between the MR-positive FCD and MR-negative FCD groups (solid line: MR-positive FCD, dashed line: MR-negative FCD).

**Fig 3:**
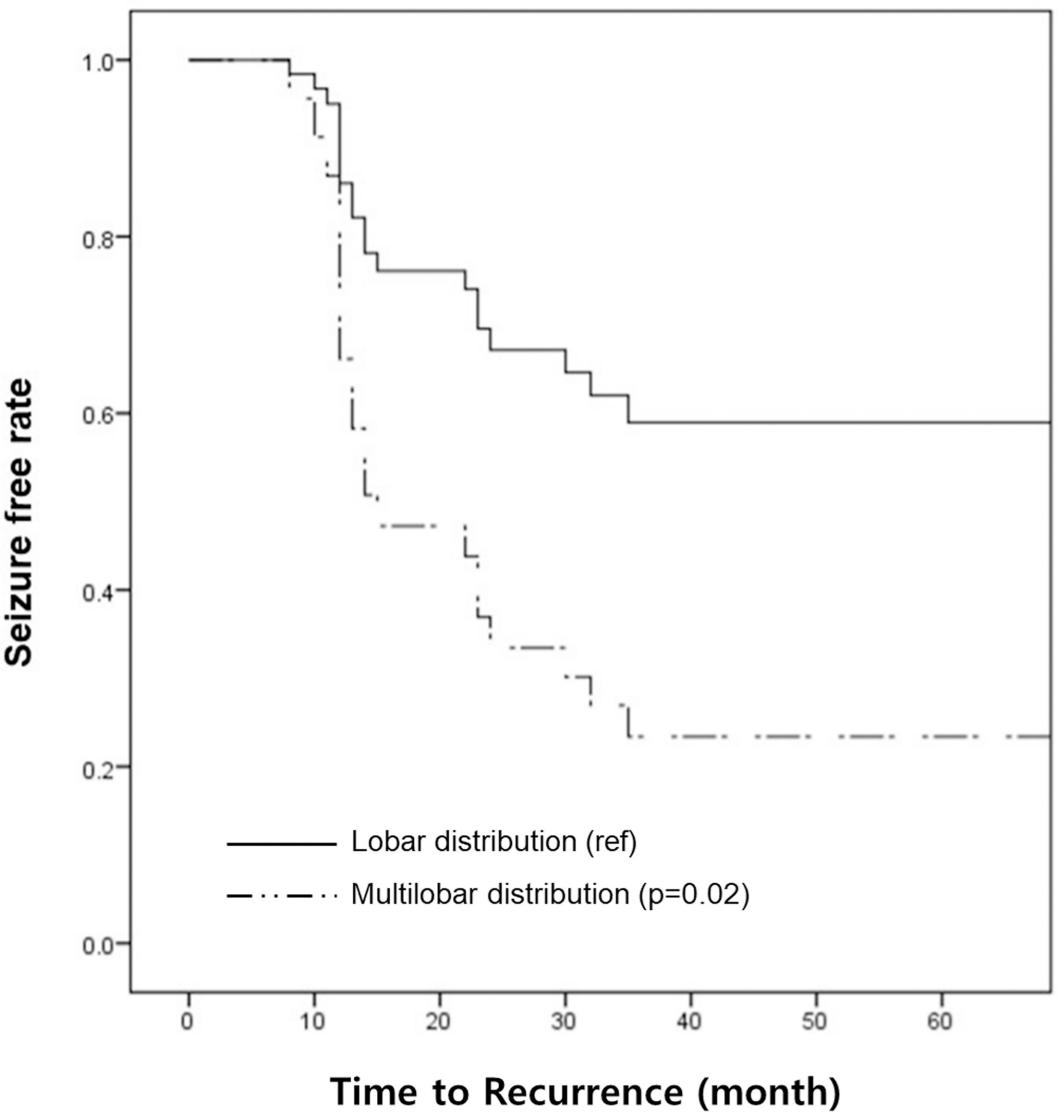
Kaplan-Meier survival curves according to SISCOM distribution in the MR-negative group. The survival curves show significantly more frequent recurrence in multilobar distribution at SISCOM finding (solid line: Lobar distribution, dashed line: Multilobar distribution).

The proportions of Engel class I (seizure-free) did not differ at two and five postoperative years between the MR-negative and MR-positive FCD groups (Table 2). Kaplan-Meier survival analysis and log-rank test did not show any differences in the seizure-free rate within a five-year follow-up period between the groups (p = 0.76) (Fig 2). In MR-negative FCD, seizure recurrence was significantly related to multilobar distribution on SISCOM (hazard ratio 2.74, p = 0.02) (Fig 3).

The good surgical prognostic factors of MR-negative FCD were evaluated by univariate analysis and multivariate logistic regression (Table 3). In the univariate analysis for two-year outcome, unilobar SISCOM distribution (odds ratio, OR 5.41, p = 0.02) and concordance of IED (odds ratio, OR 5.10, p = 0.02) was found to be good prognostic factors. In the multivariate analysis, unilobar SISCOM distribution was also identified as a good prognostic factor (OR 4.88, p = 0.02).

**Table 3:** Univariate analysis of presurgical evaluations and pathologic characteristics associated with good surgical outcomes at 2 years (Engel I) in MR-negative FCD patients.

	Engel I (n = 28)	Engel II-IV (n = 19)	*P*	Odd ratio (95% CI)
Ictal onset, unilateral	23 (82.1%)	12 (63.1%)	0.18	2.68 (0.70–10.28)
IED, concordant	17 (68.0%)	5 (29.4%)	0.02*	5.10 (1.33–19.47)
PET, unilobar	17 (60.7%)	8 (42.1%)	0.24	2.21 (0.65–6.95)
PET, concordant	17 (60.7%)	7 (36.8%)	0.14	2.64 (0.79–8.81)
SISCOM, unilobar	15 (71.4%)	6 (31.5%)	0.02*	5.41 (1.39–20.96)
SISCOM, concordant	14 (66.6%)	8 (42.1%)	0.20	2.75 (0.76–9.94)
FCD Type I	23 (82.1%)	19 (100.0%)	0.07	(-)

Data are n (%) values.

Abbreviations: IED, interictal epileptiform discharges; PET, positron emission tomography; SISCOM, subtraction ictal SPECT coregistered to MRI; FCD, focal cortical dysplasia; CI, confidence interval.

*p<0.05, independent t-test or Mann-Whitney U-test.

## Discussion

This study observed that MR-negative FCD showed 59.5% and 57.5% of seizure-free rates and MR-positive FCD showed 64.7% and 44.4% in the second and fifth years, respectively. The results of this study were comparable to those of previous studies reporting a 30–58% surgical success rate in MR-negative FCD patients with mixed age groups [13,27–29]. It is encouraging that the seizure-free rate was not different between MR-negative FCD and MR-positive FCD at the 2^nd^ and 5^th^ years, as shown in the Kaplan-Meier survival analysis and log-rank test. Moreover, unilobar SISCOM and interictal epileptiform discharges on scalp EEG were revealed to be prognostic factors for excellent surgical outcomes in MR-negative FCD patients. The importance of functional neuroimaging has been verified in previous studies [30–33]. The proportions of localizing SISCOM and FDG-PET findings did not show significant differences between the groups. However, almost all MR-negative FCD patients underwent FDG-PET or SISCOM (100% and 85%, respectively), but not in MR-positive FCD patients. This suggests that sufficient functional imaging information may lead to more precise iEEG implantation and improve surgical outcomes in MR-negative FCD patients.

Remarkably the success rate of the 5^th^ postoperative year was similar to that of the 2^nd^ year (-2.5%) in MR-negative FCD compared to MR-positive FCD (-20.3%). All MR-positive FCD patients with poor outcomes in the 5^th^ year (n = 15/27) showed non-localized cerebral perfusion in SISCOM without exception. Considering that unilobar SISCOM was a significant prognostic factor in MR-negative FCD patients, localizing or unilobar hyperperfusion may be a crucial factor to be related to long-term surgical outcomes even in MR-positive FCD patients.

The surgical outcome of FCD Type II is frequently reported to be better than that of FCD Type I [8,14,31,34]. FCD Type I is the mildest form of the FCD pathology and it is not easy to resect completely due to poorly identified lesions on MRI [1,13,14,35]. It was more frequently found in MR-negative FCD patients, thus it was not related to poor surgical outcomes. As the pathological grade was higher, more cytoarchitectural abnormalities, such as giant neurons, balloon cells, or dysplastic neurons, were found. These cellular abnormalities are more vulnerable to seizures [35].

Nevertheless, one study reported that FCD Type I had better postoperative prognosis, where the seizure-free rate in FCD Type I (65%) was superior to FCD Type II (45%). It was disclosed that distribution of FCD Type II in the extratemporal regions was significantly associated with poor surgical outcomes [36]. We also found that the percentage of extratemporal resection was much higher in MR-positive FCD patients (73.5%) than in MR-negative FCD patients (51%). In approximately one-third of MR-positive FCD patients, frontal areas were resected for epileptic foci based on iEEG monitoring. Nineteen patients of 47 MR-negative FCD remained seizure-free after five years postoperatively, and 17 of them showed FCD Type I in temporal areas. Moreover, their PET and SISCOM findings were all unilobar or concordant to epileptic foci regardless of pathology type. Only five of 15 patients with MR-positive FCD, who also had poor outcome at the 5^th^ year, had unilobar or concordant to epileptic foci results in PET and SISCOM studies. Moreover, the resection areas of the other 10 patients with MR-positive FCD with poor outcome were frontal or multiregional areas. We observed that FCD Type I was more prevalent in MR-negative FCD and their short- and long-term surgical outcomes were not different from those of MR-positive FCD. Based on the detailed analyses, surgical outcomes were more associated with localized areas of epileptic focus (temporal vs. extratemporal) rather than FCD pathology.

In this study, unilobar distribution on SISCOM and concordance of IED were significantly related to excellent surgical outcomes (Engel I) in MR-negative FCD. Moreover, the seizure-free rate in the survival analysis was related to the unilobar distribution on SISCOM exclusively in MR-negative FCD patients (hazard ratio 2.74). SPECT is associated with hemodynamic changes, and hyperperfusion of SPECT reflects the spread of adjacent cortical regions as well as the anatomic origin of epileptic discharge [37–39]. There is a controversy as to whether the hyperperfusion regions of the SISCOM agree with the actual epileptogenic zone [30,40]. Nevertheless, it is more acceptable that multifocal epileptogenic zones or rapid propagation of seizures result in multilobar distribution of SISCOM. In this study, SISCOM played a role as a prognostic factor rather than FDG-PET. In previous literature, the importance of FDG-PET was emphasized more than that of SISCOM as a prognostic factor in epilepsy surgery. The location of the epileptogenic zone and the number of SISCOM tests in this study might have accounted for this difference from the previous report. FDG-PET is known to a have high sensitivity in temporal lobe epilepsy and SISCOM in extratemporal region epilepsy. Approximately half of MR-negative FCD patients had an epileptogenic zone in the temporal areas, which is considered one of the reasons for increasing the value of SISCOM. Other studies performed SISCOM or ictal SPECT in only 50–70% of patients [1,14]. In contrast to other studies that performed SISCOM with 50–70% of subjects, 85% of MR-negative FCD patients fulfilled ictal and interictal SPECTS for SISCOM analyses, which may contribute to a more precise localization of the epileptic zones. In this study, the concordant rate of ictal EEG and IED (consistent with resection areas) on scalp EEG monitoring were 40.5% and 52.3%, respectively in MR-negative FCD. The proportions of regional or lateralized scalp ictal EEG were 59.6% and 63.8% in the MR-negative group, respectively. Some studies did not find any correlations between scalp EEG findings and surgical outcomes [1,14]. However, it was a consensus that better seizure outcome was achieved when ictal EEG or IED was concordant to the epileptogenic focus [35,41]. Non-regional IED and ictal EEG may result in incomplete resection and poor surgical outcome. We identified concordant IED as a good prognostic factor (OR, 5.10) in MR-negative FCD.

This study has several limitations. First, this was a retrospective study. We admit that a selection bias might have occurred during the selection of the patients for surgery. Physicians tend to defer surgery even in patients with intractable epilepsy if their MRIs are normal. We may have chosen the candidates who presented more affirmative data from presurgical evaluations unintentionally. Second, two different MRI equipment were used. During the 18 years study period, a 1.5T MRI apparatus was exchanged with a 3.0T apparatus. We have applied the same MRI epilepsy protocol throughout the years. It admits that the resolution of 3T MRI is definitely higher than that of 1.5T. However, it was not possible to re-confirm the absence of lesions in the MRI that were taken in 1.5T for this study because the MRI raw data of the late 90’s were discarded.

In conclusion, we observed that the short and long-term surgical outcomes of MR-negative FCD were not worse than those of MR-positive FCD patients. Good predictive factors following surgery were unilobar SISCOM distribution and concordance of interictal epileptiform discharges in MR-negative FCD patients. This study suggests that both electrophysiology and functional neuroimaging studies are necessary to obtain favorable surgical outcomes in patients with MR-negative epilepsy.

## Supporting information

S1 Dataset (XLSX)
